# U-Shaped Association Between Serum Uric Acid and Short-Term Mortality in Patients With Infective Endocarditis

**DOI:** 10.3389/fendo.2021.750818

**Published:** 2021-11-02

**Authors:** Xuebiao Wei, Bingqi Fu, Xiaolan Chen, WeiTao Chen, Zhenqian Wang, Danqing Yu, Guozhi Jiang, Jiyan Chen

**Affiliations:** ^1^ Division of Cardiology, Guangdong Cardiovascular Institute, Guangdong Provincial Key Laboratory of Coronary Heart Disease Prevention, Guangdong Provincial People’s Hospital, Guangdong Academy of Medical Sciences, Guangzhou, China; ^2^ Division of Geriatric Intensive Medicine, Guangdong Provincial Geriatrics Institute, Guangdong Provincial People’s Hospital, Guangdong Academy of Medical Sciences, Guangzhou, China; ^3^ Division of Cardiology, The First Affiliated Hospital of Shantou University Medical College, Shantou, China; ^4^ School of Public Health (Shenzhen), Sun Yat-sen University, Shenzhen, China

**Keywords:** infective endocarditis, uric acid, prognosis, U-shaped, hyperuricemia

## Abstract

**Background:**

Increased uric acid (UA) levels have been reported to be associated with poor clinical outcomes in several conditions. However, the prognostic value of UA in patients with infective endocarditis (IE) is yet unknown.

**Methods:**

A total of 1,117 patients with IE were included and divided into two groups according to the current definition of hyperuricemia (UA>420 μmol/L in men and >360 μmol/L in women): hyperuricemia group (n=336) and normouricemia group (n=781). The association between the UA level and short-term outcomes were examined.

**Results:**

The in-hospital mortality was 6.2% (69/1117). Patients with hyperuricemia carried a higher risk of in-hospital death (9.8% *vs*. 4.6%, p=0.001). Hyperuricemia was not an independent risk factor for in-hospital death (adjusted odds ratio [aOR]=1.92, 95% confidence interval [CI]: 0.92-4.02, p=0.084). A U-shaped relationship was found between the UA level and in-hospital death (p<0.001). The in-hospital mortality was lower in patients with UA in the range 250–400 μmol/L. The aOR of in-hospital death in patients with UA>400 and <250 μmol/L was 3.48 (95% CI: 1.38-8.80, p=0.008) and 3.28 (95%CI: 1.27-8.51, p=0.015), respectively. Furthermore, UA>400 μmol/L (adjusted hazard ratio [aHR]=3.54, 95%CI: 1.77-7.07, p<0.001) and <250 μmol/L (aHR=2.23, 95%CI: 1.03-4.80, p=0.041) were independent risk factors for the 6-month mortality.

**Conclusion:**

The previous definition of hyperuricemia was not suitable for risk assessment in patients with IE because of the U-shaped relationship between UA levels and in-hospital death. Low and high levels of UA were predictive of increased short-term mortality in IE patients.

## Introduction

Infective endocarditis (IE) is a rare but serious infectious disease that is defined by infection of the endocardial surface, native or prosthetic heart valves, or indwelling cardiac devices (1, 2). Although diagnostic technology and therapeutic strategies have improved significantly in recent decades, the prognosis of IE remains poor (3–5). Epidemiological data have indicated that the short-term mortality of IE is ~10% (6–8). Early identification of high-risk patients is essential for optimal therapeutic regimens.

Uric acid (UA) is the end product of purine nucleotides’ degradation and is mainly eliminated by the kidney and the intestinal tract (9). It functions as a potent antioxidant extracellularly to scavenge free radicals; however, as an intracellular prooxidant, it can disturb the bioavailability of nitric oxide in the endothelium, activate the renin-angiotensin system, stimulate the proliferation of vascular smooth muscle cells, and promote inflammation (10). Therefore, accumulating evidence suggests a J- or U-shaped relationship between UA level and prognosis (11–13).

A similar phenomenon also occurs in an infectious state. Hypouricemia has been reported to be associated with disease severity and poor prognosis in patients with coronavirus disease 2019 (COVID-19) (14). By contrast, hyperuricemia can result in high mortality rate in patients with acute respiratory distress syndrome (15). IE is also an infectious disease that is frequently complicated with cardiac dysfunction (3). Increased UA is common in the setting of heart failure because of the increased production resulting from oxidative stress and decreased excretion due to renal insufficiency (16). However, few studies have explored the prognostic value of UA in patients with IE. Here, we investigated the nature of the link between UA and an adverse prognosis in patients with IE.

## Methods

### Patient Enrolment

**Figure 1:**
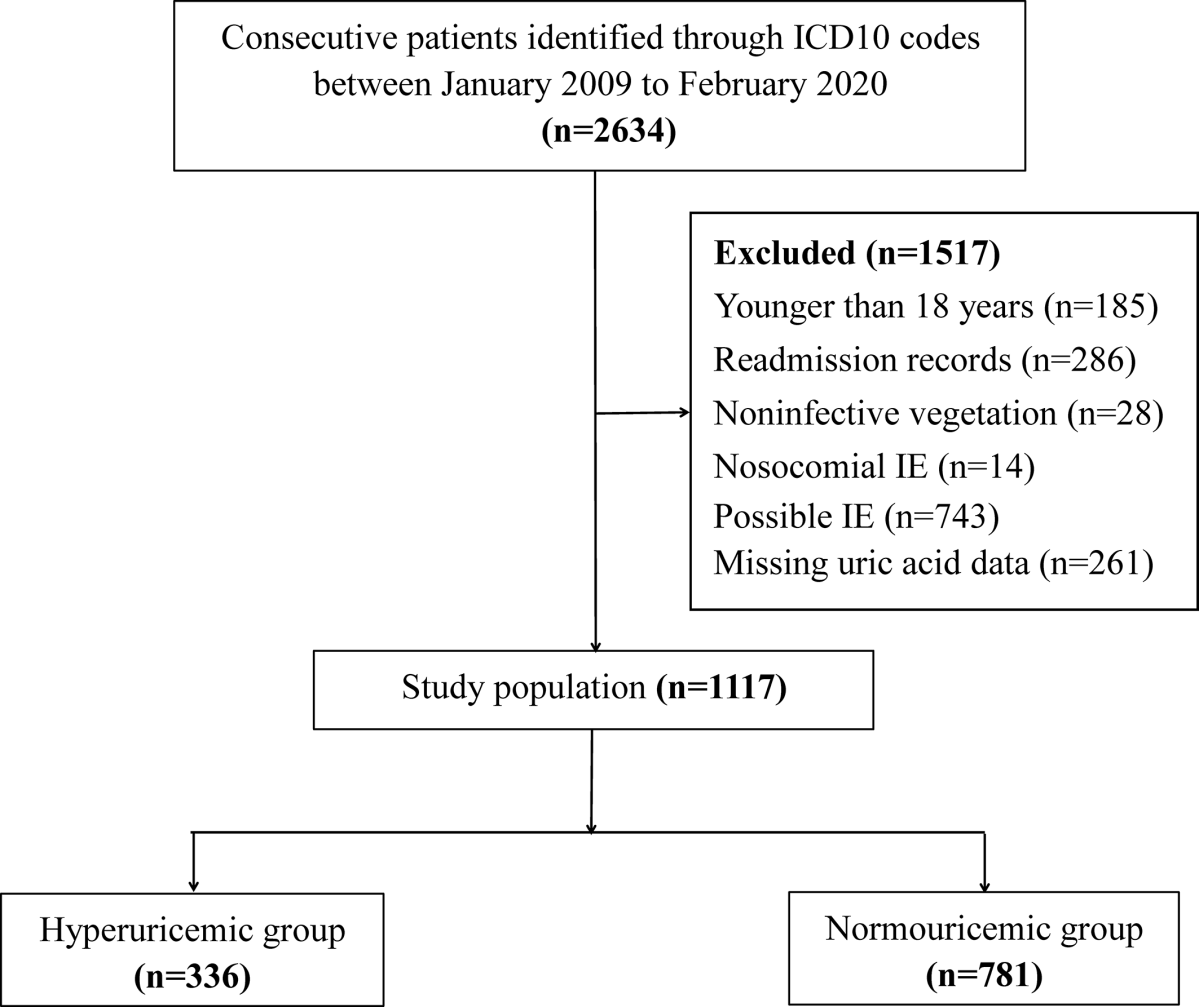
Flow chart of patient screening.

This was a retrospective study conducted in Guangdong Provincial People’s Hospital. Consecutive patients between January 2009 and February 2020 were selected from the electronic medical records according to the *International Classification of Diseases 10* codes for endocarditis: I33.0 (acute and subacute infective endocarditis), I33.9 (acute endocarditis, unspecified), and T82.6 (infection and inflammatory reaction due to cardiac valve prosthesis). IE was diagnosed by pathologic or clinical criteria based on the modified Duke criteria (17). The exclusion criteria were as follows (i) age <18 years; (ii) noninfective vegetation; (iii) nosocomial IE; (iv) possible IE (17); and (v) missing UA data. For patients who were admitted with IE more than once, only the first episode of recorded IE was included for analysis. A total of 1,117 patients were included for the final evaluation (**Figure 1**).

### Ethical Approval of the Study Protocol

The study protocol was approved (GDREC2020098) by the ethics committee of Guangdong Provincial People’s Hospital (Guangdong, China). Owing to the retrospective nature of the study, the ethics committee waived the need for written informed consent.

### Measurements and Data Collection

All participants underwent echocardiography within 24 h after hospital admission. Valve involvement and vegetations were evaluated. The left ventricular ejection fraction (LVEF) was obtained using the Simpson biplane method. Uric acid levels were measured on LX20, DXC800, or AU5800 systems (Beckman Coulter, Fullerton, CA, USA) based on a colorimetric method. A serum UA level of >420 μmol/L in men and >360 μmol/L in women was defined as hyperuricemia (10). The estimated glomerular filtration rate (eGFR) was calculated using the formula established by the Chronic Kidney Disease Epidemiology Collaboration (18). Demographics, medical history, results of laboratory tests and microbial culture, and treatment methods of the study population were collected from the electronic medical records by one researcher and checked randomly by another researcher. Clinical events were double-recorded. Inconsistent data were verified by a third researcher.

### Follow-up and Endpoints

Patients were followed-up *via* telephone for 6 months. In addition, the records for hospital readmission and outpatient-clinic interviews were reviewed for possible events. The primary endpoint was in-hospital mortality. The secondary endpoints were 6-month mortality (defined as any cause of death within 6 months after hospital admission), acute heart failure, and the need for renal replacement therapy (RRT) during hospitalization. The acute heart failure was defined as symptomatic heart failure at rest (New York Heart Association Class IV) and requiring inotropic support.

### Statistical Analyses

Normally distributed continuous data are presented as mean ± SD and were compared using the Student’s *t-*test. Non-normally distributed continuous data with are presented as the median and interquartile range and were compared using the Mann–Whitney *U*-test. Categorical data are presented as percentages and were compared using the chi-square test or Fisher’s exact test. Restricted cubic splines with three knots nested in the logistic regression analysis were used to flexibly model the association of UA with in-hospital mortality. Potential non-linearity was examined with a likelihood ratio test comparing the model with only a linear term against the model with linear and cubic spline terms. After careful visual inspection of the shape of UA’s odds ratio (OR) curves for mortality, we identified the threshold of UA at the points, if any, where risk of mortality ceased to decline or started to rise steeply, as described in previous studies (19, 20). For convenient clinical application, the nearest integer was selected. The OR and 95% confidence interval (CI) were calculated. The association between variables and the 6-month mortality were assessed by Cox proportional hazard analyses. Significant variables in the univariate regression analysis were inputted into the multivariate regression analysis. A Kaplan–Meier curve was created to evaluate the cumulative 6-month mortality in patients with different levels of UA and compared using the log-rank test. Statistical analyses were undertaken using R-software (version 3.6.2; http://www.R-project.org) and SPSS 22.0 (IBM Corporation, Armonk, NY, USA). For all analyses, p<0.05 was considered to indicate statistical significance.

## Results

### Patient Characteristics at Baseline

Among the 1,117 patients included in the present study, 336 (30.1%) had hyperuricemia. Patients with hyperuricemia were more likely to be men and have a history of hypertension, congenital heart disease, and hemodialysis than those with normouricemia. Patients with hyperuricemia also presented more usually with heart failure of New York Heart Association (NYHA) grade III/IV; had a higher body weight; higher levels of hemoglobin, fasting blood-glucose, and serum creatinine; but lower C-reactive protein (CRP) level, LVEF, and positive blood culture than those with normouricemia. The aortic valve was involved more often than the mitral valve in patients with hyperuricemia (**Table 1**).

**Table 1:** Baseline characteristics.

Clinical variables	Hyperuricemic group (n = 336)	Normouricemic group (n = 781)	P value
Age (year)	46.5 ± 15.4	44.7 ± 15.7	0.070
Female gender, n (%)	81 (24.1)	264 (33.8)	0.001
Body weight (kg)	59.7 ± 12.7	55.5 ± 10.4	<0.001
Comorbidities, n (%)			
Hypertension	71 (21.1)	110 (14.1)	0.003
Diabetes	33 (9.8)	51 (6.5)	0.056
Rheumatic heart disease	60 (17.9)	118 (15.1)	0.250
Congenital heart disease	120 (35.7)	224 (28.7)	0.020
History of hemodialysis, n (%)	6 (1.8)	3 (0.4)	0.042
Prosthetic valve, n (%)	24 (7.1)	41 (5.2)	0.215
NYHA Class III/IV heart failure, n (%)	143 (42.6)	212 (27.1)	<0.001
WBC (×10^9^/L)	9.7 ± 4.3	9.9 ± 4.3	0.504
Platelet (×10^9^/L)	187.8 (124.6,269.0)	207.0 (127.8,278.8)	0.133
Hemoglobin (g/L)	108.3 ± 25.0	102.8 ± 20.6	0.001
Fasting blood-glucose (mmol/L)	5.4 ± 1.9	5.2 ± 1.4	0.150
CRP (mg/L)	16.8 (7.1,39.4)	35.3 (14.0,67.2)	<0.001
Serum creatinine (umol/L)	92.0 (77.0,133.0)	70.7 (57.8,85.9)	<0.001
eGFR<60 ml/min/1.73m^2^	109 (32.4)	65 (8.3)	<0.001
LVEF (%)	63.8 ± 8.0	65.5 ± 6.8	0.001
Vegetation, n (%)	323 (96.1)	757 (96.9)	0.495
Vegetation present, n (%)			
Aortic valve	180 (53.6)	277 (35.5)	<0.001
Mitral valve	163 (48.5)	486 (62.2)	<0.001
Aortic+Mitral valve	47 (14.0)	76 (9.7)	0.037
Other sites	27 (8.0)	70 (9.0)	0.614
Blood culture positive, n (%)	176 (52.4)	517 (66.2)	<0.001
Surgical treatment, n (%)	249 (74.1)	556 (71.2)	0.319
Embolic events, n (%)	57 (17.0)	160 (20.5)	0.172
Length of hospital stay (days)	32 (19,44)	35 (20,46)	0.072
In-hospital events, n (%)			
Acute heart failure	33 (9.8)	56 (7.2)	0.133
Renal replacement treatment	33 (9.8)	32 (4.1)	<0.001
Death	33 (9.8)	36 (4.6)	0.001

NYHA, New York Heart Association; CRP, C-reactive protein; eGFR, estimated glomerular filtration rate; LVEF, left ventricular ejection fraction.

### UA and In-Hospital Events

**Figure 2:**
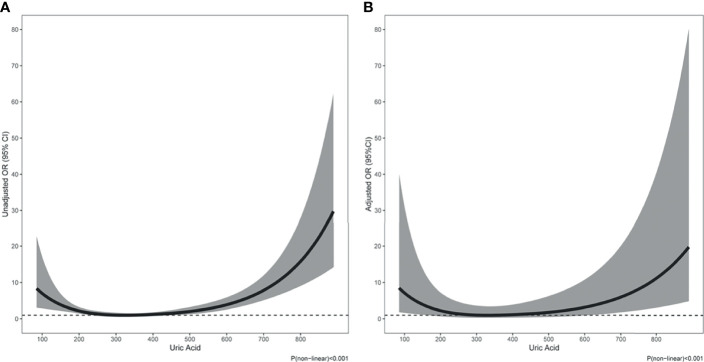
The association of uric acid with in-hospital death **(A)** Unadjusted; **(B)** Adjusted.

**Figure 3:**
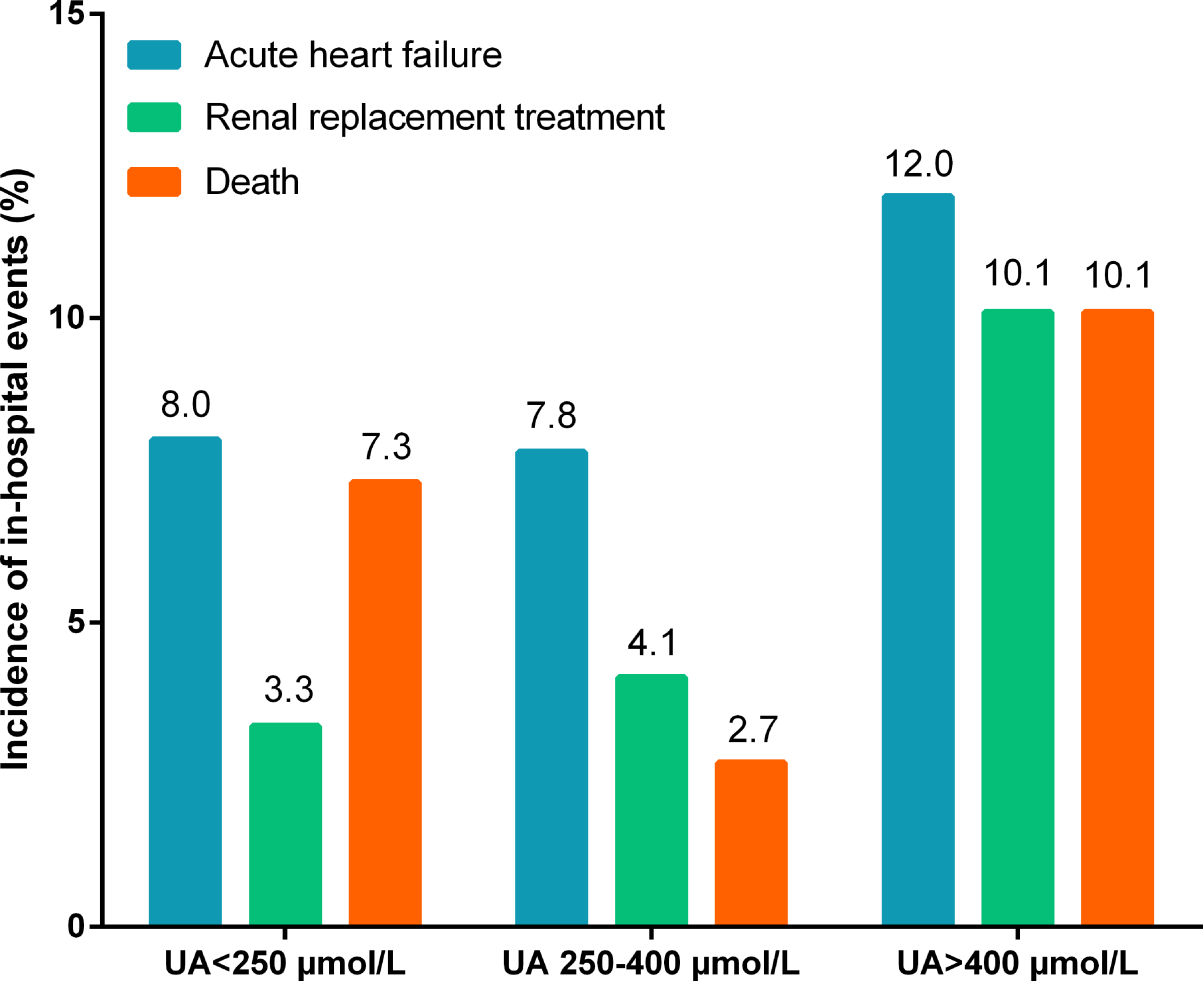
Incidence of in-hospital events according to different uric acid levels.

At the time of hospitalization, 69 (6.2%) patients died, 89 (8.0%) suffered acute heart failure, and 65 (5.8%) required RRT. The incidence of in-hospital death (9.8% *vs*. 4.6%, p=0.001; **Table 1**) and RRT (9.8% *vs*. 4.1%, p<0.001; **Table 1**) were significantly higher in patients with hyperuricemia than those with normouricemia. However, hyperuricemia was not an independent risk factor for in-hospital death after adjustment for age, hypertension, diabetes, prosthetic valve, NYHA Class III/IV, white blood cell (WBC), platelet <150×109/L, anemia, fasting blood-glucose, logCRP, eGFR<60 mL/min/1.73 m^2^, LVEF, aortic valve vegetation, mitral valve vegetation, and surgical treatment (adjusted OR=1.92, 95%CI: 0.92-4.02, p=0.084; **Table 2**). A U-shaped trend was observed between the UA level and in-hospital death (p<0.001; **Figure 2A**). The in-hospital mortality was low in patients with UA in the range of 250–400 μmol/L (**Figure 2A**). Patients with UA>400 μmol/L suffered the highest risk of in-hospital death (10.1% *vs*. 2.7% *vs*. 7.3%, p<0.001; **Figure 3**) and RRT (10.1% *vs*. 4.1% *vs*. 3.3%, p<0.001; **Figure 3**).

**Table 2:** Univariate and multivariate logistic regression analysis for in-hospital mortality.

	Hyperuricemia (UA>420 μmol/L in men and >360 μmol/L in women)		UA>400 μmol/L *vs*. 250-400 μmol/L		UA<250 μmol/L *vs*. 250-400 μmol/L	
	OR (95% CI)	p	OR (95% CI)	P	OR (95% CI)	p
**In-hospital death**						
Model 1: unadjusted	2.25 (1.38-3.68)	0.001	4.08 (2.13-7.82)	<0.001	2.87(1.40-5.86)	0.004
Model 2: multivariate adjusted*	1.92 (0.92-4.02)	0.084	3.48 (1.38-8.80)	0.008	3.28(1.27-8.51)	0.015

UA, uric acid; OR, odds ratio; CI, confidence interval; NYHA, New York Heart Association; CRP, C-reactive protein; eGFR, estimated glomerular filtration rate; LVEF, left ventricular ejection fraction.

*Adjusted variables included age, hypertension, diabetes, prosthetic valve, NYHA Class III/IV, WBC, platelet <150×10^9^/L, anemia, fasting blood-glucose, lgCRP, eGFR<60ml/min/1.73m2, LVEF, aortic valve vegetation, mitral valve vegetation and surgery treatment.

Multivariate logistic regression analysis showed that compared with UA in the range of 250–400 μmol/L, UA>400 μmol/L (adjusted OR=3.48, 95%CI: 1.38-8.80, p=0.008; **Table 2**) and <250 μmol/L (adjusted OR=3.28, 95%CI: 1.27-8.51, p=0.015; **Table 2**) were significantly associated with in-hospital death after adjustment for confounding factors. In addition, The U-shape relationship was similar to the univariable model without adjustment (p<0.001; **Figure 2B**).

### UA and 6-Month Mortality

**Figure 4:**
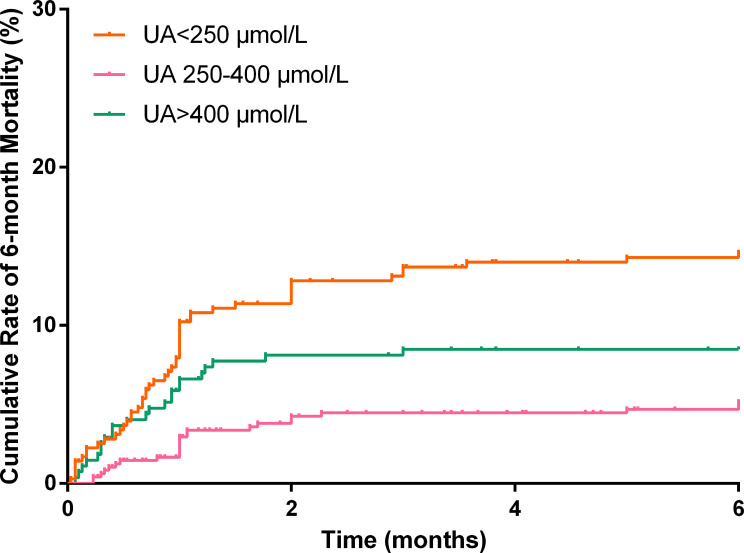
Cumulative rate of 6-month mortality according to different uric acid levels.

In total, 1,037 (92.8%) patients completed the 6-month follow-up, and the 6-month mortality rate was 9.5%. The Kaplan–Meier curve indicated that the cumulative 6-month mortality was significantly higher in patients with UA>400 μmol/L (log-rank test=22.4, p<0.001; **Figure 4**). In addition, UA>400 μmol/L (adjusted hazard ratio [HR]=3.54, 95%CI: 1.77-7.07, p<0.001; **Table 3**) and <250 μmol/L (adjusted HR=2.23, 95%CI: 1.03-4.80, p=0.041; **Table 3**) were independent risk factors for 6-month mortality in the multivariate Cox survival analysis.

**Table 3:** Univariate and multivariable Cox regression analysis for 6-month mortality.

Clinical variables	Univariate analysis		Multivariable analysis	
	HR (95% CI)	P	HR (95% CI)	P
Age	1.04 (1.02-1.05)	<0.001	1.01 (0.99-1.03)	0.603
Female gender	0.68 (0.43-1.09)	0.109		
Weight	1.00 (0.99-1.02)	0.712		
Hypertension	1.60 (1.00-2.55)	0.049	0.86 (0.45-1.63)	0.639
Diabetes	2.34 (1.35-4.06)	0.002	1.41 (0.63-3.18)	0.406
Rheumatic heart disease	2.01 (1.28-3.15)	0.002	0.91 (0.46-1.81)	0.790
Congenital heart disease	0.98 (0.64-1.50)	0.916		
History of hemodialysis	1.38 (0.19-9.93)	0.747		
Prosthetic valve	4.50 (2.73-7.43)	<0.001	2.87 (1.36-6.04)	0.006
NYHA class III or IV	4.53 (2.98-6.88)	<0.001	3.33 (1.85-5.98)	<0.001
WBC	1.08 (1.04-1.11)	<0.001	1.02 (0.97-1.07)	0.471
Platelet <150×10^9^/L	2.55 (1.72-3.80)	<0.001	1.83 (1.10-3.05)	0.020
Anemia	2.90 (1.59-5.31)	0.001	2.15 (0.96-4.79)	0.061
Fasting blood-glucose	1.30 (1.22-1.40)	<0.001	1.12 (1.02-1.23)	0.022
lgCRP	3.75 (2.21-6.36)	<0.001	1.64 (0.86-3.14)	0.135
eGFR<60ml/min/1.73m^2^	3.90 (2.60-5.85)	<0.001	1.00 (0.97-1.03)	0.841
LVEF	0.96 (0.94-0.98)	<0.001	2.41 (1.30-4.45)	0.005
Aortic valve vegetation	2.80 (1.85-4.24)	<0.001	0.99 (0.56-1.74)	0.968
Mitral valve vegetation	0.45 (0.30-0.67)	<0.001	0.21 (0.12-0.35)	<0.001
Surgery treatment	0.19 (0.13-0.29)	<0.001	2.87 (1.36-6.04)	0.006
UA, μmol/L				
250-400	1 [Reference]	-	1 [Reference]	-
<250	1.72 (0.97-3.05)	0.062	2.23 (1.03-4.80)	0.041
>400	3.01 (1.86-4.90)	<0.001	3.54 (1.77-7.07)	<0.001

NYHA, New York Heart Association; CRP, C-reactive protein; eGFR, estimated glomerular filtration rate; LVEF, left ventricular ejection fraction.

## Discussion

To our knowledge, this is the first study to explore the prognostic role of serum UA levels in patients with IE. We discovered a U-shaped relationship between the UA level and in-hospital mortality. The current definition of hyperuricemia was not an independent risk factor for in-hospital death in patients with IE. Both UA>400 μmol/L and <250 μmol/L were independently associated with in-hospital and 6-month mortality, which could be considered an optimal threshold for predicting poor prognosis in IE patients.

In our analysis, 30.1% IE patients had hyperuricemia, which could be attributed to a high incidence of renal and cardiac insufficiency in IE. In healthy individuals, two-thirds of daily UA is excreted by the kidneys and the remaining one-third is eliminated through the intestinal tract. In renal dysfunction, the renal excretion of UA reduced and the intestinal excretion is compromised by impairment of UA transporters, resulting in a high serum UA levels (21). With respect to heart failure, increased production of UA because of increased xanthine-oxidase activity as well as decreased renal excretion of UA because of renal hypoperfusion together contribute to an increase in the UA level (16). In addition to clinical manifestations in these two conditions, hyperuricemia could serve as an important indicator for unfavorable outcomes. Hyperuricemia plays a part in the progression of chronic kidney diseases (13, 22), as well as potentiating the risk of acute kidney injury during hospitalization (23, 24). In patients with acute/chronic heart failure, an increased UA level is believed to correlate independently with increased short-term and long-term mortality as well as re-hospitalization (25–27). However, the clinical importance of hyperuricemia in IE is still unclear.

We showed that hyperuricemia was a risk factor for in-hospital mortality, but its significance was lost after multivariate regression analysis. A U-shaped relationship between the UA level and in-hospital mortality for IE patients was discovered, which was consistent with previous studies (11–13). It was believed that the previous definition of hyperuricemia (UA>420 μmol/L in men and >360 μmol/L in women) was not sufficiently valid for predicting clinical outcomes in certain pathological conditions. The J- or U-shaped relationship was gradually accepted. A recent study conducted by Chen et al. included 1,854 patients with COVID-19 infection and showed a U-shaped association between UA and composite outcomes (28). Their cut-off values were ≥423 µmol/L and ≤278 µmol/L, which was close to our findings.

This U-shaped relationship might be explained by the dual antioxidant and prooxidant effect of UA in inflammatory conditions. In humans, >50% antioxidant capacity in blood plasma comes from UA (29). In addition, UA has been shown to be effective in preventing viral infection by enhancing T-cell responses and secretion of type-I interferons (30, 31). However, UA levels have been observed to decrease continuously in people with infectious diseases (32, 33). Consistently, the UA level was lower in patients with a positive blood culture in our study. Hypouricemia has a prognostic value to some extent. An extremely low level of UA diminishes the antioxidant capacity of plasma in severe sepsis and indicates a poor outcome (34).

Despite its protective effect, an extremely high UA level may signify more harm than benefit during severe inflammation. Our results suggested that an increased UA level was associated with a poor outcome in IE. The latter is a microbial infection on the endocardial surface, wherein the spread of pathogenic organisms into the bloodstream can trigger a systemic inflammatory response syndrome. The incidence of sepsis in 294 IE patients was nearly 30% in a study conducted by Krajinovic et al. (35). Chuang et al. showed that the UA level was positively correlated with the Acute Physiology and Chronic Health Evaluation (APACHE) score in patients with sepsis, which supported its role in reflecting illness severity (36). Septic patients with hyperuricemia tend to require greater vasopressor support (37). Lee et al. found that in patients with acute respiratory distress syndrome, the mortality was higher in cases with normal-to-high UA level than in patients with a low UA level (15). The possible underlying mechanism might be explained by the excessive prooxidant effect in a hyperuricemia environment that leads to endothelial dysfunction, increased activity of xanthine oxidase, increased oxidative stress, inappropriate activation of the renin–angiotensin–aldosterone system, impaired renal autoregulatory response, and release of proinflammatory chemokines (38).

Our study has some limitations. First, this was a single-center study with a small sample size (1,117 cases with 69 events). We used package “pwr2ppl” (version 0.2.0) in R software to calculate power for logistic model. Our study included 1117 cases with 69 events that had 82% to 99% power for detecting ORs of 1.50 to 1.90 at an alpha level of 0.05, with 6% in-hospital mortality and 0.20 correlation between UA and other covariates. Therefore, we think that the sample size is relatively powerful to detect the association between UA and mortality. Second, this study was retrospective in nature. Although we adjusted for most potential confounding factors in the multivariate analysis, residual factors might have affected the results. Third, the UA level is different in male and female patients, but the newly identified cut-off value of UA was not distinguished based on because of the small study cohort. Last, telephone interviews, hospital-readmission records, and outpatient clinic interviews were employed during the follow-up period, but some patients showed poor compliance.

## Conclusions

Low and high levels of UA were independent risk factors for in-hospital and 1-year mortality in patients with IE. The previous definition of hyperuricemia (UA>420 μmol/L in men and >360 μmol/L in women) was not suitable for risk stratification because of the U-shaped trend between the UA level and adverse outcomes. UA>400 or <250 μmol/L might be more valuable predictors of outcome than the previous cut-offs, especially for infectious diseases.

## Data Availability Statement

The raw data supporting the conclusions of this article will be made available by the authors, without undue reservation.

## Ethics Statement

The study protocol was approved (GDREC2020098) by the Ethics Committee of Guangdong Provincial People’s Hospital (Guangdong, China). Written informed consent for participation was not required for this study in accordance with the national legislation and the institutional requirements.

## Author Contributions

DY and JC contributed to the conception or design of the work. XW, BF, XC, and WC contributed to the acquisition or interpretation of data for the work. ZW and GJ contributed to statistical analysis. XW, BF, and XC drafted the manuscript. DY and JC critically revised the manuscript. Everyone gave final approval and agreed to be accountable for all aspects of the work ensuring integrity and accuracy. All authors contributed to the article and approved the submitted version.

## Funding

This study was supported by grants from the National Natural Science Foundation of China (grant no. 82002014), Natural Science Foundation of Guangdong Province (grant no. 2021A1515010107), Science and Technology Projects of Guangzhou (grant no. 201903010097), and Guangdong Provincial Key Laboratory of Coronary Heart Disease Prevention (grant no. 2017B030314041). The funders had no role in the study design, data collection and analysis, decision to publish, or preparation of the manuscript.

## Conflict of Interest

The authors declare that the research was conducted in the absence of any commercial or financial relationships that could be construed as a potential conflict of interest.

## Publisher’s Note

All claims expressed in this article are solely those of the authors and do not necessarily represent those of their affiliated organizations, or those of the publisher, the editors and the reviewers. Any product that may be evaluated in this article, or claim that may be made by its manufacturer, is not guaranteed or endorsed by the publisher.
